# Behavioral effects on the offspring of rodent mothers exposed to Tetrahydrocannabinol (THC): A meta-analysis

**DOI:** 10.3389/fpsyg.2022.934600

**Published:** 2022-08-26

**Authors:** Simón Ramírez, Gonzalo Miguez, Vanetza E. Quezada-Scholz, Luis Pardo, Felipe Alfaro, Felipe I. Varas, Mario A. Laborda

**Affiliations:** ^1^Department of Psychology, Universidad de Chile, Santiago, Chile; ^2^Department of Social Sciences and Humanities, Universidad de Aysén, Coyhaique, Chile

**Keywords:** drugs, Tetrahydrocannabinol (THC), rodents, behavior, offspring, mothers

## Abstract

Pre and perinatal administration of Tetrahydrocannabinol (THC) in rodents and their offspring has many effects that have been studied using different methods that have not been integrated using quantitative methods. The effect of THC administration on behavior can be better understood by meta-analytic techniques. We examined whether there is an overall effect on the behavior of the offspring when THC is administered to mothers. Eligibility criteria included experiments using an experimental design with a control group without THC, in which THC is administered to mothers during pregnancy and lactation in rodents, and in which at least one type of behavioral (locomotor, emotional or cognitive) measurement in the offspring was implemented. Cohen’s *d* was obtained for each study, then each individual study was weighted, and moderator analysis was performed. Analysis was performed using fixed and random effect models, and the heterogeneity was assessed by calculating Qb, *I*^2^ and the prediction interval. Furthermore, 3 sub-meta-analyses were carried out according to the type of behavior. The general analysis determined a low weighted effect size of THC on the behavior of the offspring, moderated by type of rat strain. The sub-meta-analyses showed a medium effect for cognitive effects of THC in the offspring, and a low effect on locomotor activity and emotional behavior. In addition, publication bias was not detected. More research is needed to contribute to the understanding of the effect of THC exposure on offspring.

## Introduction

There is increasing use of cannabis and its derivatives in the general population, including mothers who consume it during or after pregnancy (Volkow et al., 2019), which raises the need to consider the consequences of mothers’ drug consumption on their offspring as a research concern. One way to study the behavioral effects in subjects descended from mothers with experience with cannabis intake is through the use of animal models, specifically rodents. These allow better experimental control of the amount of substance consumed or administered and its association with the effects on behavior, controlling for the influence of other intervening variables. Some reviews have described these effects, but they have not integrated them quantitatively, so it is relevant to determine it using meta-analytical techniques.

The use of cannabis is frequent in the global population. In 2018, 192 million people around the world used it at least on one occasion, of which one-third were women (United Nations Office against Drugs and Crime [UNODC], 2020). It has been reported that in the U.S. pregnant population, the prevalence of past-month cannabis use between 2002–2003 and 2016–2017 has increased from 3.4 to 7.0% (Volkow et al., 2019). The high prevalence of its consumption in this type of population generates an increasing interest to investigate the possible effect of cannabis during pre and perinatal stages.

Marijuana or cannabis *Sativa* sp. has more than 70 cannabinoid components. One of these is Δ-9-Tetrahydrocannabinol (THC), which is highly psychoactive. THC is a fat-soluble molecule that is able to cross the placenta and stay stored in milk, making it a molecule capable of interacting with both the fetus and the neonate. Furthermore, due to its psychoactive characteristics, THC concentration has increased by 20% in plants in the last 30 years (Lafaye et al., 2017; Stuyt, 2018). The possible consequences that the THC molecule can exert *via* the placenta and lactation in the behavior of the offspring has generated a great interest in understanding its effects. This interest has driven researchers to realize several experiments with diverse results, so its quantification in an integrated manner must be considered.

For instance, in adolescent and adult humans the use of cannabis during pregnancy has been shown to generate neurocognitive alterations in their offspring (e.g., Rubino et al., 2008; Castellanos-Ryan et al., 2016; Sagar and Gruber, 2018). Several developmental effects have also been reported, such as stunted growth and decreased weight at birth (e.g., Linn et al., 1983; Fried et al., 1984, 1999; Cornelius et al., 1995). Other reported effects are problems in visual (Fried et al., 1998) and visuospatial working memory (Smith et al., 2006), verbal reasoning (Fried and Watkinson, 1990), and impulsivity and aggression (Leech et al., 1999; Goldschmidt et al., 2000), among others.

In animal models, studies using cannabis extract have been conducted using mainly rodents, particularly rats, due its easy handling and superior behavioral characteristics (i.e., cognitive). In pregnant rats, behavioral and physiological effects of consumption on the offspring have been reported since the 1970s. As in humans, several effects of maternal consumption have been reported, such as reduction in birth weight and effects on learning (e.g., Gianutsos and Abbatiello, 1972; Borgen et al., 1973; Uyeno, 1973; Vardaris et al., 1976; Abel et al., 1980, 1981; Brake et al., 1987). To date, different reviews have synthesized this literature, qualitatively analyzing both the neurochemical and behavioral effects on the offspring exposed pre- and perinatally to THC (e.g., Abel, 1980; Navarro et al., 1995; Ferraro et al., 2009; Schneider, 2009; Campolongo et al., 2011; Higuera-Matas et al., 2015), but none of them have integrated the possible effect quantitatively. These reviews show a consensus in the way of reporting the behavioral or dependent variable, separating them into locomotor activity, emotional, and cognitive behavior, according to the tests, tasks, and measurement carried out in each study analyzed. This division or categorization of behavior is based on the classification observed in mammals, and social insects mainly.

Reviews show that the three behavioral variables mentioned above are used as dependent variables in studies of the effects of THC on offspring. It is worth mentioning that there is variability in the operationalization of such behavior, particularly concerning emotional behavior (Kremer et al., 2020) and there are no clear limits in the definitions. It should also be noted that the definition of behavior is broad and depends on the theoretical framework used. Conceptually, we will take emotional behavior or emotional reactivity as the social-affective life of the animal. Locomotor activity or behavior is related to motor coordination and/or free movement, and cognitive behavior related to attention, memory, and learning. At the operational level, the dependent variables of the different experiments indicate the prevalence of one of these three aspects of behavior in the execution of a task. For example, in experiments in which it emphasized cognitive behavior or the evocation of emotions, the type of task performed was fundamental for the classification of the dependent variable.

The effects on locomotor activity in subjects descended from mothers with experience with THC are varied; hyperactivity has been reported, with the administration of either synthetic THC or WIN 55,212-2 (WIN; Mereu et al., 2003) and natural THC (Borgen et al., 1973; Rubio et al., 1995), while no significant effects were observed with hashish extract (Navarro et al., 1994). On the other hand, some studies reported a reduction in locomotor activity under the administration of natural THC (Fried, 1976).

The literature focused on the effects of emotional behavior also reports dissimilar results. For example, an increase in isolation-induced ultrasonic vocalizations (USVs, which are indicative of anxiety) has been observed (Trezza et al., 2008) as well as a decrease (Antonelli et al., 2005). Similarly, in social interaction behavior, both a reduction (O’Shea et al., 2006; Trezza et al., 2008) and an increase (Newsom and Kelly, 2008) have been reported. Regarding depression, measured by forced swimming test, no differences were reported between the control and experimental groups (Newsom and Kelly, 2008). The evidence describes, as well as locomotor behavior, heterogeneous results in subjects who descended from mothers with exposure to THC. Therefore, the direction of emotional behavior toward anxiolytic or anxiogenic effects cannot be concluded in ontogenetic development (Bambico et al., 2010) and in the development of offspring in rodents.

In cognitive behavior, the direction of the effect is most clear. The administration of WIN in mothers induces in the offspring a disruption in the working memory retention in an inhibitory avoidance task (Mereu et al., 2003). In addition, it is reported a disruption in short-term olfactory memory in a social discrimination task (Campolongo et al., 2007). Furthermore, under the maternal administration of WIN, learning in the offspring is deficient in active avoidance tasks (Antonelli et al., 2005). In this line, in acquisition of avoidance tasks, prenatal exposure to THC in low doses (0.15 mg/kg) does not show differences with control subjects but weakens consolidation and reverse learning during juvenile and adult stages in males, but not females are reported (Silva et al., 2012). In turn, in attentional tests deterioration is reported under the previous preparation (Silva et al., 2012).

Moreover, as we already mentioned, the literature varies in the types of behaviors, but also differ in protocols executed, which mainly vary in the route or *via* of administration, the strain, the concentration or dose of THC, and the types of tests implemented. For example, the acute administration of cannabinoids can generate anxiolytic, anxiogenic or biphasic effects (Viveros et al., 2005; Moreira and Lutz, 2008). The same occurs in male offspring under novelty reactivity tests but not in maze tests (Navarro and Rodriguez de Fonseca, 1998). Other studies show a decreasing trend in behavior or emotional reactivity under a WIN treatment at low concentrations (Antonelli et al., 2005) and an increase under a THC treatment at a medium concentration (Trezza et al., 2008).

It is also known that the *via* in which the drug is administered could be of importance in order to show the effects on behavior, for example, in self-administration models the same effects have not been found in the same doses given by experimenters (Kirschmann et al., 2017). A factor that has been proven from 1970s, but not used in all protocols is the sex of the subjects. In drug studies where males and females are reported, sexual differences are found. For example, female rats explore more than males in risky associative learning tasks (Jolles et al., 2015) and in aversive taste conditioning tasks (Hempel et al., 2017), they even respond differentially to prenatal THC exposure in anxiety, cognition and locomotor tests (Silva et al., 2012; Higuera-Matas et al., 2015). From this perspective it is essential to include sex variable related to the administration of THC, however, given the few studies it was chosen not to treat it in the quantitative analysis. Under this background, the different factors of the protocols used should be considered to understand the behavioral differences of pre and perinatal THC administration.

According to the relevance that THC has on the behavior of the offspring that comes from mothers with THC experience, and from previous descriptive analyzes, our main objective in this meta-analysis is to measure the integrated effect that the prenatal and perinatal administration of THC could have on the behavior of the offspring, as well as to establish differential effects of this administration that can be explained by different mediators.

## Materials and methods

The present meta-analysis was carried out following the recommendations of the Journal Article Reporting Standards for Quantitative Research in Psychology: The APA Publications and Communications Board Task Force Report (Borenstein et al., 2009; Cooper, 2017; Appelbaum et al., 2018; Lefebvre et al., 2021).

### Searching strategy

Studies were identified following a web search of three databases: Scopus, PubMed, and the Web of Science. Reports published in any year were included. The searching terms were reviewed by two experts in the field of cognition and drug tolerance with a Ph.D Degree. In addition, reference lists of selected reports were parsed as a complementary search strategy of relevant reports. As another complementary strategy, associations of researchers related to the topic were queried for unidentified reports.

The PubMed thesaurus vocabulary was used to select search keywords. The terms “marijuana,” “THC” and “cannab*” were chosen for the independent variable. An OR operator was used between each term. For time of administration, the following terms were used: “descend*,” “mums,” “pups,” “dads” “baby,” “perinatal,” “prenatal,” “epigenetic,” “phylogenetic,” “dad,” “lactancy,” “pregnancy,” “fetus,” “fetal,” “breeding,” “gestation,” “conception,” “offspring,” “young,” “paternal,” “maternal,” “daughter,” “son,” “father” and “mother,” and between each term an OR operator was incorporated. To specify the sample, the terms “rat,*” “rod,*” “mice” and “mouse” were used, using an OR operator between each term. Each group of terms were linked with an AND operator. The “*” symbol functions as a wildcard operator in all search engines, and the same search strategies were used in the three search engines.

### Eligibility criteria and selection of studies

The articles that met the following eligibility criteria were selected: (1) studies where THC administration (natural or synthetic, without combination with other drugs) occurred during pregnancy or lactation in rats, (2) studies with an experimental design including a control group without THC, (3) studies that measured locomotor, emotional or cognitive behavior in the offspring, (4) studies that have used subjects with a pathology, abnormal behavior and/or have been genetically manipulated, (5) or studies that did not report sufficient information for the effect size calculation.

Article screening proceeded as follows: for criterion 1, title and abstract were read. For criterion 2, the abstracts, method and results were read. For criteria 3 and 4 the abstracts and methods were read. For criterion 5 abstracts and methods were read. The selection was made independently by two researchers (i.e., a graduate and an undergraduate student from the University of Chile) who were trained by the experts mentioned above. The studies were selected by one researcher to later be corroborated by the other.

### Data collection

Data was collected by the same researchers who selected the studies. The unit of analysis (UA) was each individual experiment contained in each research report. The task used in each UA was classified into either locomotor activity, emotionality or cognitive behavior. For instance, as locomotor activity, tasks such as number of crossings in open fields, immobility, mobility, and motor coordination were included. As emotional behaviors were considered tasks that mainly measure anxiety, social interaction, play and depression in open fields and elevated plus mazes. As cognitive behavior was defined, measurements focused on learning, memory, and attention tasks. UA were included according to the categorization of locomotor, emotional and cognitive behavior as a dependent variable. The direction of the effect of THC in the subjects was calculated according to whether the locomotor behavior had a diminishing, sedative or hypoactive (negative direction) or an increase, stimulating or hyperactive effect (positive direction). Emotional behavior was similarly classified, while for cognitive behavior, the classification was based upon whether the subjects showed an ability to learn and retain learning (i.e., memory), that is, an increase in cognitive ability (positive) or a decrease (negative) in relation to the control group. It is worth noting that social interaction tests were included in the emotional behavioral variable, which are classified as socio-emotional, given the limitation in the literature regarding this topic, in which the correlation between emotional reactivity and social interaction is not clear. For instance, it has been reported that an increase in emotional reactivity is related to less social interaction and more social anxiety (Campolongo et al., 2011). In this meta-analysis, on the contrary, we will include greater social interaction as greater socio-emotional reactivity.

Regarding the treatment of each UA, in the article that included multiple UAs, each one was analyzed separately. In UA that reported more than two levels in the manipulation of the independent variable, an average of the doses of THC administered was used for coding (see section “Moderator Analysis” below). When a UA presented more than one control group, they were averaged and compared with the experimental group, and the number of subjects was counted as the sum of both. In the case that UA reported daily administration, the amount of dose reported during the experimental procedure was calculated, this was done by multiplying the dose per day by the number of days of administration. In the case that the day of the end of pregnancy was not reported, this was estimated as gestational day (GD) 20. In repeated measures designs (e.g., different evaluation days), the responses were averaged, and an effect size was calculated.

When two or more measures were reported in a UA, the effect size of each measure was calculated using the combination of separated variances. In cases in which the UA reported different measures of the dependent variable, for example, a locomotion measure with an emotional one, we proceed as follows: (1) it was categorized as mixed, and an effect size was calculated for each measure and then combined and reported as a single effect size. (2) Also, they were separated to determine the orientation of the effect in each sub-meta-analysis for each dependent variable (i.e., locomotor, emotional, and cognitive). When more than one task was reported in a measure and in an UA, the effect sizes were combined and reported as a single effect size.

For UA that did not report sufficient statistics to calculate the effect size, but did report information mainly contained in graphs, the WebPlotDigitizer tool (Rohatgi, 2019) was used to extract exact values from the figures. In this way, it was possible to extract the necessary information for the calculation of Cohen’s *d* (means and standard error or SEM). Cohen’s *d* can be positive or negative, indicating an increase or decrease of the experimental group from the control, respectively. Following this, effect sizes calculated were transformed to reflect a negative impact of THC in the experimental group, when appropriate. For example, where immobility was tested, it was reported as an increase of immobility, meaning a positive effect size. To transform the effect size, it was multiplied by –1, resulting in a negative effect size, reflecting the diminished mobility. This transformation of the effect sizes allowed for proper combination with meta-analytic techniques. In the example about immobility, we multiplied by –1, because it indicates sedative or hypoactive behavior, this was done with the aim of standardizing the measure toward the direction of the effect. The coding was done by the two researchers separately. Each experiment in each article was coded as a separate UA. These codes included characteristics of the experiments and parameters of each experimental phase.

### Moderator analysis

This analysis can show sources of variability, therefore it is done to differentiate the contribution of the different categorical moderators. The moderators and their categories were: type of THC (natural or synthetic), strain used (Sprague-Dawley, Wistar, Long Evans), and amount of exposure to the drug reported during the experiment or lifetime dose (high and low). To facilitate analysis of the latter moderator, its code was treated in a dichotomous way. The sample of UA was divided into two halves according to dose, then a new variable was introduced, classifying each half as high dose (i.e., upper half) and low dose (i.e., lower half). Route or *via* of administration (intraperitoneal, intravenous, oral, subcutaneous), type of behavior (locomotor, emotional, cognitive, mixed) was also coded. A reliability analysis of the codes used in the moderators was carried out between the two researchers, which was reported using Cohen’s Kappa. To resolve discrepancy both coders met to compare results and resolve them.

### Methods used to control the risk of internal validity of primary studies

To analyze the internal validity of the primary studies and the possible presence of sources of systematic error or biases, we followed the recommendations of O’Connor and Sargeant (2014) and Higgins et al. (2021). First, the maintenance methods and protocols were analyzed to ensure proper care and control of animals, to detect possible biases in baseline differences between groups. The performance biases, which analyze the conditions of maintenance of the subjects in the groups except in the experimental phase, were checked by reading the maintenance protocols and the presence of an ethics committee’s approval. Detection biases, which can occur when there are systematic differences between treatment groups in the way the outcome is assessed, were analyzed by calculating effect sizes independently. For attrition biases, referring to systematic differences in withdrawals of subjects from treatment groups, the methods and results were checked.

### Measurements and data analysis

The data extracted together with the number of subjects in each group of each UA were entered into an online effect size calculator (Wilson, n.d.). Once all the effect sizes of each UA were computed, the individual weights of each effect size were calculated, based on the number of experimental subjects and the variance participating in each UA (Borenstein et al., 2009; Cooper, 2017). Standardized mean differences were then obtained for each experiment and then synthesized using a weighted mean effect size method under a fixed and random effects model, to assess the robustness of the results using different statistical assumptions. Thus, the variance of the Cohen’s *d* of this meta-analysis is estimated through the within-study variance for both models. The analysis using a fixed effect model operates under the assumption that the data reflect a single average effect size despite the variation in procedures and tasks, while the random effects model analysis is based on the assumption that the effect sizes in the analyzed studies represent a random sample (Borenstein et al., 2009) of which the selected effect sizes are representative. Subsequently, 95% confidence intervals (CI) were calculated around the estimate of the means of the integrated effect size. Once all effect sizes were calculated the homogeneity was analyzed using the statistic of Hedges and Olkin (1985) called Qt. The *I*^2^ statistic was also calculated; *I*^2^ indicates which proportion of the total variance of the effect sizes is due to the variance between the studies. Tau squared (*T*^2^) and its predictability interval were also calculated. For the analysis of categorical moderators, we used the Qb and Qw tests of homogeneity. For this assessment, the values of the degrees of freedom of each group (k) were determined, and then compared with the chi-square critical values. A non-significant value of Qb shows that the means are homogeneous. To assess publication bias (both in the main meta-analysis and in the sub-meta-analyses) we tested the asymmetry of the distribution of effect sizes using the Trim and Fill method (Duval and Tweedie, 2000) and the Egger test (Egger et al., 1997). Analyzes and calculations were performed using Microsoft Excel according to Cantor (1996); Borenstein et al. (2009), and Cooper (2017) for the integrated effects size and through the use of comprehensive software Meta-Analysis Student Professional version 3.3070 to corroborate and to do the following analysis. Both funnel and forest plot were created using the latter software.

## Results

### Study selection

**FIGURE 1:**
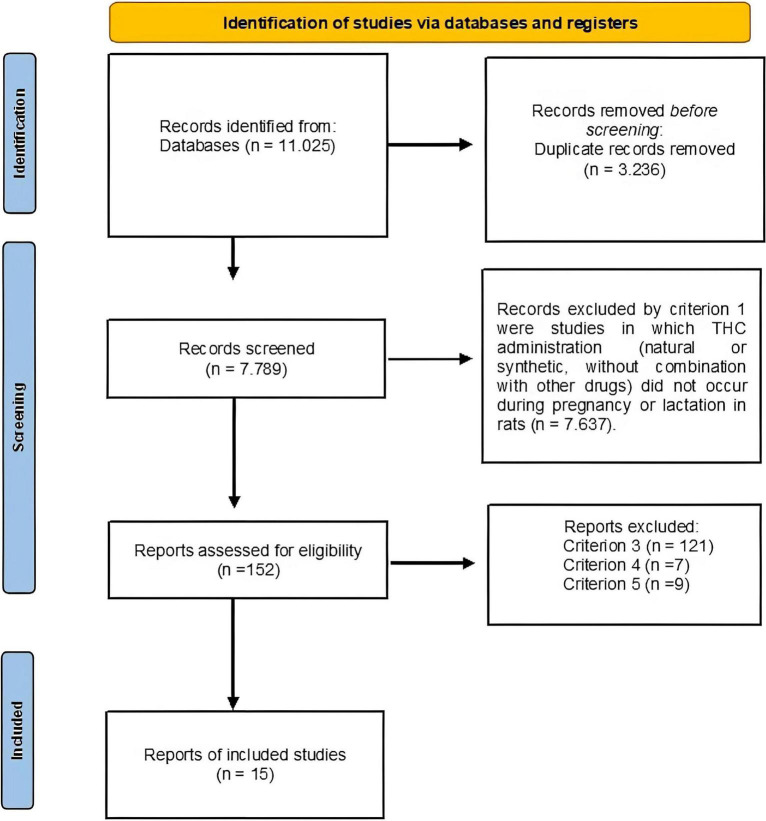
Flowchart for the search of articles and its selection. The boxes in the left columns indicate the number of articles selected. Each arrow indicates the detection procedure. The boxes in the right column show the number of articles excluded due to each eligibility criteria.

Database search results are summarized in Figure 1, which also illustrates the screening process. A total of 11,025 articles were compiled, of which 3,236 were duplicates After removing duplicates, a total of 7,789 remained. The complement strategies did not find articles that were not contained in the searching. Subsequently, of these 7,789 studies, 7,637 were discarded because THC was not used during the pre and perinatal stages in rodent mothers (i.e., criterion 1); of the remaining 152 studies, 121 were discarded because they did not present a locomotor, emotional or cognitive behavioral measure (i.e., criterion 3); 9 were discarded because they did not report enough data to calculate the effect size (i.e., criterion 4), and 7 were discarded because the subjects were either a genetic strain to model a pathology, or were genetically manipulated (i.e., criterion 5). Thus, the final sample consisted of 15 studies, from which 28 UAs were extracted.

### Characteristics of the studies

The UAs included are summarized in Table 1. Regarding the independent variable, the majority of UAs used THC (18), and then WIN (10 UA). Rats were used in all the included UAs, mostly Wistar strain (20 UA), then Sprague-Dawley (4 UA), and finally Long-Evans (4 UA). The administration was mostly by oral route (14 UA), then subcutaneous injection (11 UA), and intravenous injection (3 UA). Tests for emotional behavior were included in 8 UA, mixed also in 8 UA, cognitive in 6 UA, and locomotor activity in 4 UA. Cohen’s Kappa or inter coder agreement was 0.82, discrepancies were resolved as mentioned in Moderator Analysis.

**TABLE 1:** Study characteristics.

N	First author	Cannabinoid receptor agonist	Doses	Period	Lifetime dose	Route	Behavior	Sex and strain	Test age	Result
1	Antonelli et al. (2005)	WIN	0.5 mg/kg	GD5 to GD20	8 mg/kg	S.C.	Cognitive, Emotional	Male Wistar	PND 6, 8, 10, and 12	Decreased performance in homing behavior (learning and memory), Decreased separation-induced ultrasonic vocalization
2	Bara et al. (2018)	WIN	0.5 mg/kg	GD5 to GD20	8 mg/kg	S.C.	Emotional, Mixed cognitive emotional	Male–Female Wistar	PND ≥ 90	Decreased playing and social behavior, No change in discrimination task, No change in time spend in open arms EPM
3	Borgen et al. (1973)	THC	10 mg/kg	GD10 to GD12	30 mg/kg	S.C.	Locomotor activity	Male Wistar	PND9, 13, 17, and 21	Increased locomotor activity
4	Bortolato et al. (2006)	WIN	0.5 and 1 mg/kg	GD5 to GD20	8 mg/kg and 16 mg/kg	S.C.	Cognitive	Male Long-Evans	PND40, 60, and 80	No effect on prepulse inhibition
5	Campolongo et al. (2007)	THC	5 mg/Kg	GD15 to PND 9	80 mg/kg	Oral	Cognitive	Male Wistar	PND81	Decreased smell memory in a social discrimination task.
6	Drazanova et al. (2019)	THC	5 mg/Kg	GD15 to PND 9	80 mg/kg	Oral	Mixed emotional cognitive	Male Sprague-Dawley	PND180	Decreased time in social interaction, Decreased discrimination index of a new object
7	Moreno et al. (2003)	THC	0.1 0.5, and 2 mg/Kg	GD5 to PND 24	3.9, 19.5, and 78 mg/kg	S.C.	Locomotor activity	Male-female Wistar	PND70	Decreased locomotor activity
8	Mereu et al. (2003)	WIN	0.5 mg/Kg	GD 5 to GD20	8 mg/Kg	S.C.	Locomotor activity	Male Wistar	PND12, 40 and 80	Increased locomotor activity
9	Navarro and Rodriguez de Fonseca (1998)	THC	1, 5, and 20 mg/Kg	GD5 to PND 24	30, 150, and 600 mg/kg	Oral	Mixed Locom-Emot, Emotional	Male-female Wistar	PND15, 20, 30, 40, and 70	Increased Locomotor activity, Decreased emotional reactivity (more time in open arms), Increases emotional activity, No change in spontaneous locomotor activity
10	Navarro et al. (1994)	THC	5 mg/kg	GD5 to PND24	150 mg/kg	Oral	Emotional, Locomotor	Male-Female Wistar	PND20, 30, 40, and 70	Increased emotional reactivity, rearing increase, Increased emotional reactivity, reactivity to novelty increase, No change in locomotor activity
11	Newsom and Kelly (2008)	THC	4 mg/kg	GD1 to PND10	40 mg/kg	S.C.	Emotional	Male Long-Evans	PND93, 97, and 99	Increased emotional reactivity (Decreased time spent in the center of the open arena), Increased emotional activity (increases sniffing behavior), No change in forced swimming test
12	Rubio et al. (1995)	THC	5 mg/kg	GD 5 to PND24	225 mg/kg	Oral	Mixed Locom-Emot, Emotional	Male -female Wistar	PND70	Increased emotional reactivity rearing, sniffing, grooming, Increased locomotor activity, Decreased emotional reactivity (Increased open arm entries and open arm time in the EPM)
13	Shabani et al. (2011)	WIN	0.5 mg/kg	GD 5 to GD20	8.5 mg/kg	S.C.	Mixed Locom-Emot	Male Wistar	PND22, 36 and 50	Decreased coordination time in a rotarod, Decreased sniffing and increased grooming, No effect in time spent in the center of the open field, Decreased Locomotor activity
14	Silva et al. (2012)	THC	0.15 mg/Kg	GD 1 to GD 21	3.15 mg/kg	I.V.	Cognitive	Male -Female Sprague-Dawley	PND22 and 23 PND45 and 46 PND60	Decreased passive avoidance retention (Memory), Decreased active place avoidance (behavioral plasticity), Decreased Attention (more trails to complete a attentional task)
15	Trezza et al. (2008)	THC	2.5 and 5 mg/Kg,	GD15 to PND9	40–80 mg/kg	Oral	Mixed Locom-Emot, Emotional	Male Wistar	PND12	Increased separation-induced ultrasonic vocalization, Decreased emotional behavior (social interaction), Increased emotional behavior (playing)

Specific characteristics and their categories of each study with their respective UA. It is divided into treatment of mothers, and the measurement and the subjects’ behavioral tasks. S.C, subcutaneous; I.V, Intravenous injection; PND, Postnatal day; GD, Gestational day; THC, Delta-9-tetrahydrocannabinol; WIN, WIN 55,212-2; EPM, elevated plus maze.

### Effect size

**FIGURE 2:**
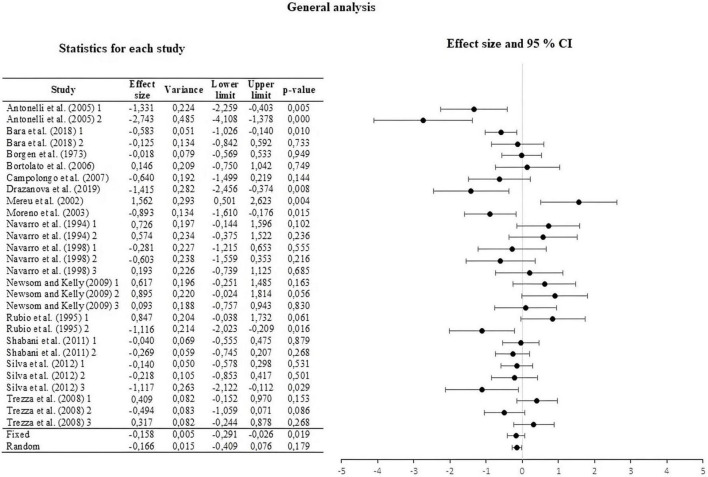
Forest plot of the effect sizes and estimated confidence intervals of all Unit of Analysis (UA). For the integrated effect size Cohen’s d was used. The lines indicate the effect sizes of each UA and its corresponding 95% confidence intervals. The last two lines represent the result under a fixed and random effect model, respectively. The lower and upper limits, the *p*-values and the variance of each UA are reported.

We encoded the effect size of the 28 UA using Cohen’s *d*. The summary of the measures and their forest plot can be seen in Figure 2. The weighted effect size under a fixed effect model was *d* = –0.158, 95% CI [–0.291, –0.026]. Under a random effect model, the effect size was *d* = –0.166, 95% CI [–0.409, –0.076]. The results of both analyzes show a low effect of THC administered to mothers on the behavior of the offspring. Similar effect sizes of both models suggest that the statistical assumption does not affect the conclusion about the magnitude of the effect size. The test of heterogeneity for the fixed effect model reported by Hedges & Olkin’s Q was 82,459, which was higher than the reported critical value of chi-square (*df* = 27); thus, allowing a rejection of the null hypothesis, while *I*^2^ = 67,256, that is, a medium heterogeneity level. Finally, *T*^2^ = 0.267 and *T* = 0.517, with a prediction interval of [–1.23, 0.91].

### Moderators analysis

**FIGURE 3:**
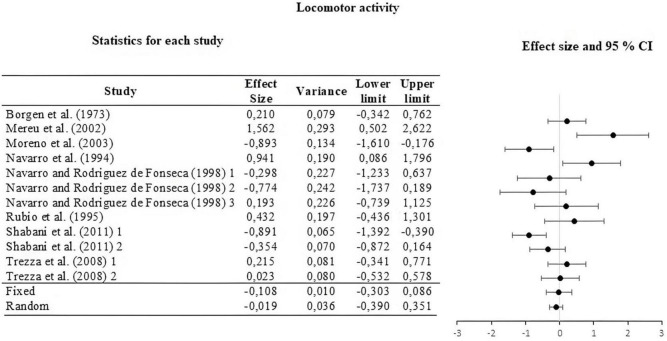
Forest plot of the effect sizes and estimated confidence intervals of the UAs that evaluated locomotor activity. For the integrated effect size Cohen’s d was used. The lines indicate the effect sizes of each UA and its corresponding 95% confidence intervals. The last two lines represent the result under a fixed and random effect model, respectively. The lower and upper limits and the variance of each UA are reported.

**FIGURE 4:**
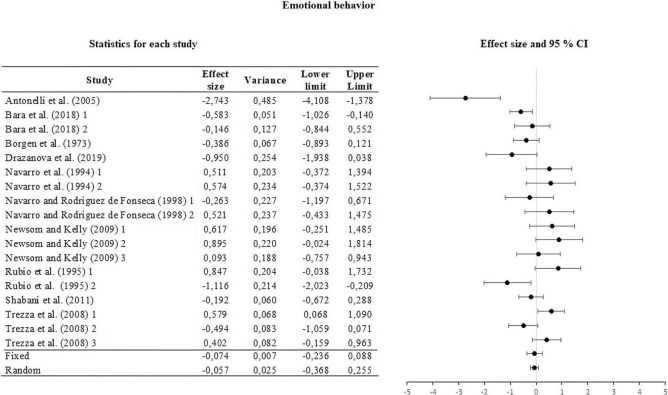
Forest plot of the effect sizes and estimated confidence intervals of the UAs that evaluated emotional behavior. For the integrated effect size Cohen’s d was used. The lines indicate the effect sizes of each UA and its corresponding 95% confidence intervals. The last two lines represent the result under a fixed and random effect model, respectively. The lower and upper limits and the variance of each UA are reported.

**FIGURE 5:**
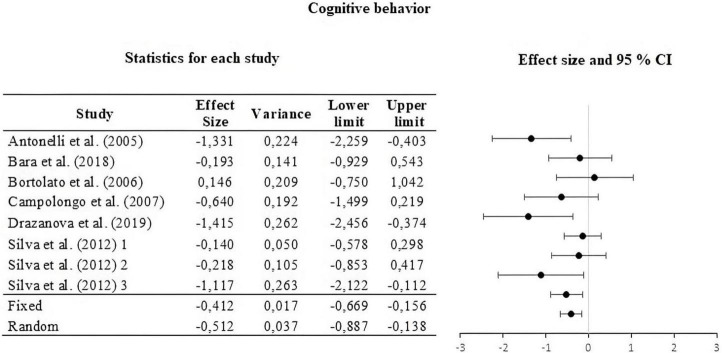
Forest plot of the effect sizes and estimated confidence intervals of the UAs that evaluated cognitive behavior. For the integrated effect size Cohen’s d was used. The lines indicate the effect sizes of each UA and its corresponding 95% confidence intervals. The last two lines represent the result under a fixed and random effect model, respectively. The lower and upper limits and the variance of each UA are reported.

For this, the weight of the five categories that contributed to the calculation of the effect size was analyzed and calculated. The information is summarized in Table 2. The moderators used were strain, type of THC administered, amount of dose administered, route or *via* of administration, and type of task. The results of the moderator analysis were: strain Qb = 7.042; *df* = 2 which is greater than the critical value (5.99), type of THC Qb = 3.419; *df* = 1 which is less than the critical value reported (3.84), dose during life Qb = 0.029; *df* = 1; that is less than the reported critical value (3.84), administration route Qb = 0.746; *df* = 2 that is less than the reported critical value (5.99) and task Qb = 6.324; *df* = 3 which is less than the critical value (7.81). According to Qb, a difference is only seen between the means of the effect sizes in the strain categories. The three sub-meta-analyses under the fixed and random effect model are presented in Figures 3–5. For locomotor activity (12 UA) the weighted effect size under a fixed effect model was *d* = –0.106, 95% CI [–0.303, 0.086] and random *d* = –0.019, 95% CI [–0.389, 0.351], for emotional behavior (18 UA) under a fixed effect model it was *d* = –0.074, 95% CI [–0.223, 0.088] and random *d* = –0.057, 95% CI [–0.368, 0.254], and for cognitive (8 UA) under a fixed effect model *d* = –0.412, 95% CI [–0.669, –0.156] and random *d* = –0.512, 95% CI [–0.887, –0.137].

**TABLE 2:** Summary of moderator analysis.

Category	Size effect			95% IC		Null test	Heterogeneity test d		Critical Q
	k	d	Se	L.I.	L.U.	Z	Qb	*df*	Z
**Strain**									
Wistar	20	–0.182	0.081	–0.339	–0.024	–2.225	7.042	2	5.99
Sprague-Dawley	4	–0.384	0.165	–0.076	–0.061	–3.329			
Long Evans	4	0.271	0.190	–0.102	0.644	1.424			
**Drug type**									
THC	18	–0.045	0.091	–0.242	0.133	–0.498	3.419	1	5.84
WIN	10	–0.297	0.101	–0.494	–0.507	–2.943			
**Amount of doses life**									
High	13	–0.118	0.109	–0.332	0.095	–0.618	0.029	1	5.84
Low	15	–0.184	0.086	–0.353	–0.015	–4.502			
**Administration *via***									
Subcutaneous	11	–0.174	0.106	–0.383	–0.035	–1.636	0.746	2	5.99
Oral	14	–0.105	0.101	–0.303	–0.094	–1.033			
Intravenous	3	–0.274	0.173	–0.613	0.066	–1.580			
**Behavior**									
Emotional	11	–0.334	0.119	–0.577	–0.112	–2.289	6.324	3	7.81
Locomotor	3	–0.066	0.206	–0.470	0.029	–0.322			
Cognitive	5	–0.271	0.152	–0.569	–0.061	−1786			
Mixed	9	–0.038	0.111	–0.180	0.238	0.341			

Each moderator group has its respective effect size. Qb and the critical Q are reported according to critical values of the chi-square table. Qb greater than the critical values represents that the null hypothesis is rejected. L.I., Lower limit; K, number of groups in the subcategory; d, weighted effect size; se, standard error; CI, confidence interval; Qb, statistical test homogeneity; df, degrees of freedom.

### Evaluation of the internal validity of the primary studies

For selection bias, we did not detect differences in the factors that could affect UA baselines, so we suggest that there is a low risk of this bias. In addition, this possible bias was controlled by eligibility criteria when establishing that the included UAs did not include within subject designs and also was controlled for random assignment to groups. In performance biases, we found a 100% use of animals kept under standard experimental conditions for both control and experimental groups, but 25% of the studies do not report following an ethical protocol, in this sense we can detect clearly the presence of this bias. On the other hand, we found a low risk of detection bias, since the calculations were carried out independently of the reported statistics. We also found a low risk in attrition bias since the loss of animals from the groups was minimal when analyzing all UA. Finally, using the same group of subjects to measure different behavioral tasks (Mixed Category) could have an interaction effect due to the measurement of the dependent variable of one task over the other, which constitutes a risk for internal validity.

### Publication bias

**FIGURE 6:**
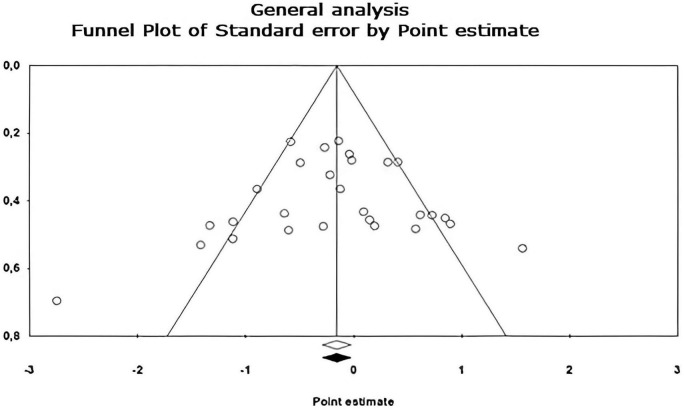
Funnel plot of publication bias created using the Trim and Fill method (Duval and Tweedie, 2000) shows an estimate of the effect sizes of all UAs. Data obtained from the experiments are shown in white. No data points were imputed.

**FIGURE 7:**
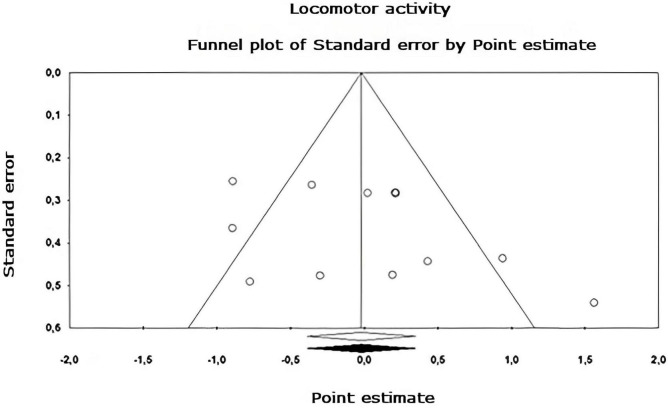
Funnel plot of publication bias for the locomotor activity sub-metaanalysis, created using the Trim and Fill method (Duval and Tweedie, 2000). It shows an estimate of the effect sizes of all UAs. Data obtained from the experiments are shown in white. No data points were imputed.

**FIGURE 8:**
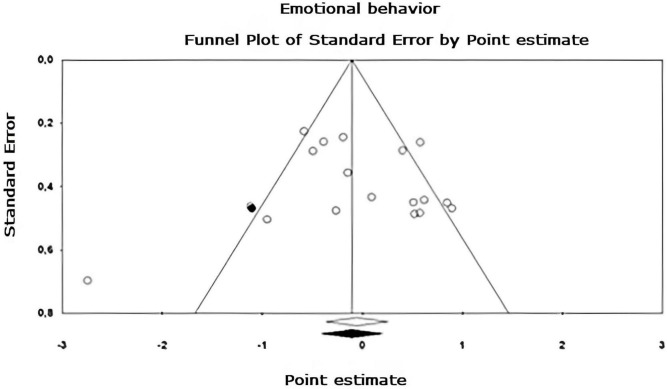
Funnel plot of publication bias for the emotional behavior sub-metaanalysis, created using the Trim and Fill method (Duval and Tweedie, 2000). It shows an estimate of the effect sizes of all UAs. Data obtained from the experiments are shown in white and the imputed data point is shown in black.

**FIGURE 9:**
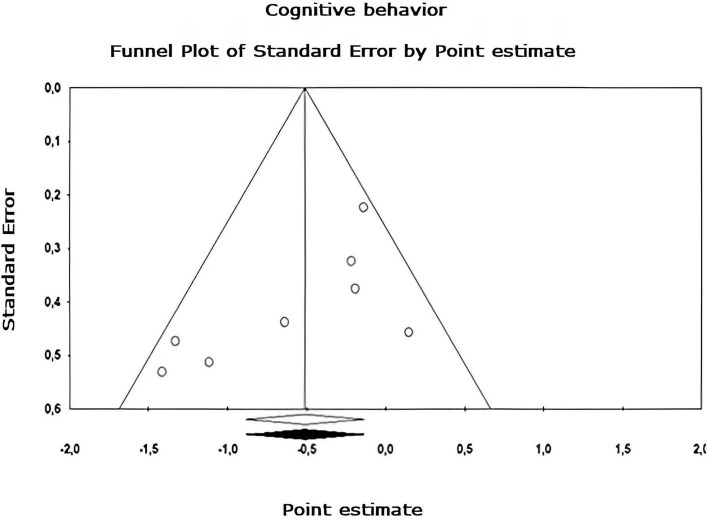
Funnel plot of publication bias for the cognitive behavior sub-metaanalysis, created using the Trim and Fill method (Duval and Tweedie, 2000). It shows an estimate of the effect sizes of all UAs. Data obtained from the experiments are shown in white. No data points were imputed.

Egger’s asymmetry test (Egger et al., 1997), reports an intersection of –0.320, 95% CI [–2.643, 2.003], with *t* = 0.283, *df* = 26. The *p*-value of 1 tail (recommended, Borenstein et al., 2009) was 0.390 and the 2-tailed *p*-value was 0.780. As there is no significance, there is no publication bias. Using the Trim and Fill method, the estimation of the effect size under the fixed effect model for the combined studies was *d* = –0.158, 95% CI [–0.291, –0.026], these values do not change. Under the random effect model, the effect size was *d* = –0.167, 95% CI [–0.409, 0.076], which also remains unchanged. A Trim and Fill Funnel Plot that indicates no asymmetry is shown in Figure 6. Publication biases of each sub-meta-analysis (Figures 7–9), show that only emotional behavior is imputed at one point. The imputed point analysis indicates that the effect size according to the fixed effect model, for the combined studies was *d* = –0.073, 95% CI [–0.236, 0.088]. Using Trim and Fill, the imputed point estimate was *d* = –0.105, 95% CI [–0.264, 0.055] and for the random effect model it was *d* = –0.057, 95% CI [–0.369, 0.255], the imputed point estimate is *d* = –0.106, 95% CI [–0.416, 0.203], which does not modify the values, thus, publication bias is not observed.

## Discussion

The present meta-analysis measured the integrated effect of prenatal and perinatal administration of THC to mothers on the behavior of their offspring using a fixed and random effect model. The main finding is that there is a low magnitude of the effect that the administration of THC to mothers have on the behavior of offspring, measured both for a fixed and random effect model according to 28 UA analyzed. With this result we mean that the decrease in behavior (i.e., locomotor, emotional, and cognitive) in descendants of mothers who were administered THC, is of low magnitude. The heterogeneity detected was medium. The CI did not reach zero in the case of the fixed effect model, not so under the random effect model that did. It should be noted that given the prediction interval, it is observed that the range from a psychological sense goes from the decrease to the increase in behavior. In this way we cannot determine the orientation in which the behavior changes, only that its integrated effect is low. Regarding the results according to the two models, the orientation and magnitude of the effect are consistent and do not depend on the statistical decision made to integrate the data, being these generalizable.

In the integrated UA we detected a low level of risk of the analyzed biases, which were mainly controlled by the eligibility criteria. In this sense, there is consistency in the way of performing the procedures between the studies. In publication biases, we did not observe publication bias, since the studies are symmetrically distributed around the mean. Thus, we cannot say that there was a limitation in the search or that there are possible missing effect sizes caused by a search limitation or by data censoring by the researchers (Cooper, 2017).

To differentially analyze the three types of behavior (i.e., dependent variable groups), three sub-meta-analyses were carried out. The cognitive task measures ordinally yielded a larger mean effect size in relation to the other two types, this measure is in line with the previous descriptive reviews where this type of studies reports a difference with the control subjects and not with tasks of an emotional nature and locomotor activity (Schneider, 2009; Campolongo et al., 2011; Higuera-Matas et al., 2015).

In emotional behavior, a low magnitude effect was found with a CI that reached zero. In the description of the UA, we observe effects with opposite orientation in the tasks. In this way, we can find that in anxiety tasks measured in elevated plus maze, it can generate an increase in emotional reactivity (Trezza et al., 2008) and a decrease in it (Rubio et al., 1995). The same is reported in emotional behavior measured under UVs (Antonelli et al., 2005; Trezza et al., 2008) and in social interaction (Newsom and Kelly, 2008; Bara et al., 2018). We can conclude that exposure to natural or synthetic THC in pre and perinatal stages, alters the emotional behavior of the offspring in opposite orientations with a low integrated effect.

A low ordinal effect was also found in locomotor activity. Given its CI, this data is not reliable. This behavior is also in line with the previous descriptive reviews, where opposite and neutral results are reported, some oriented toward sedative or hypoactive effects (Navarro and Rodriguez de Fonseca, 1998; Moreno et al., 2003; Shabani et al., 2011) and stimulant or hyperactive (Borgen et al., 1973; Navarro et al., 1994; Mereu et al., 2003). Given the studies analyzed, we can say that THC affects or alters the behavior of the offspring in an unclear way regarding the orientation of the effect, which, integrated, turns out to be of low magnitude. In the description (see Table 1) we include the results of sexual dimorphism, although our interest was not to establish or discriminate between the sex of the subjects, it is known the importance of the sexual differences observed in the behavior of the subjects studied. For example, hyperactivity is observed in female rats and not in males (Navarro and Rodriguez de Fonseca, 1998; Moreno et al., 2003). In emotional behavior we can see the effect of sexual dimorphism even more pronounced, for example we can observe that the increase in social interaction behaviors only occurs in males but not in females (Bara et al., 2018). Similarly, in light-dark emergency tests, an effect occurs only in males and not in females (Navarro and Rodriguez de Fonseca, 1998). In the future, any study that attempts to establish the effect on the behavior of subjects descended from mothers who were administered THC, should take into account both males and females in their experimental designs.

Our results determined that cognitive behavior has a clearer orientation than previous types of behavior. Specifically, subjects lose cognitive abilities relative to controls. One of the reasons why the literature reports that it could affect behavior directly is the weight of newborns, under this paradigm we can observe that there is no difference in the weight of subjects born in treatment and controls (Navarro et al., 1994; Mereu et al., 2003; Moreno et al., 2003; Antonelli et al., 2005; Campolongo et al., 2007; Newsom and Kelly, 2008; Trezza et al., 2008; Silva et al., 2012; Drazanova et al., 2019), on the other hand, in other studies there is a decrease (Shabani et al., 2011) that fades over time (Bortolato et al., 2006), so it is another mechanism or mechanisms that explains the results. The literature reports that prenatal exposure to THC alters some aspects of executive function in rats descended from mothers with exposure to THC, which is correlated with the modification of the glutamatergic transmission system (Mereu et al., 2003; Antonelli et al., 2005; Castaldo et al., 2007; Bara et al., 2018) which is fundamental for learning and memory tasks (i.e., cognitive), as well as for synaptic plasticity. In addition, executive function is represented by the activity of neurons located in the prefrontal cortex in which glutamate transmission is key in local communication in that cortex. In Antonelli et al. (2005) and Mereu et al. (2003) we can find that THC and WIN affect glutamatergic cortical neurotransmission in the offspring, which reinforces that idea. Other reports of direct effects show that THC decreases working memory and inhibits the concentration of extracellular acetylcholine in the hippocampus (Lichtman and Martin, 1996; Nava et al., 2000).

Another factor that may explain our result is THC is related to a reduction of the expression of GLUT1 (main glucose transporter in oocyte, embryo and placenta) likely to cause proper fetal development (Natale et al., 2020; Kuzma-Hunt et al., 2021; Lee and Hardy, 2021), in that line it is reported that deficiency in GLUT 1 is related with cognitive impairment (De Giorgis et al., 2019). THC also is demonstrated to decrease fetal blood space and increase maternal blood space which may be indicative of a damaged nutrient transport between mother and the fetus (Natale et al., 2020).

Another indicator related to cognitive behavior is the BDNF factor, which is mainly responsible for regulating synapses. Adolescent rats administered THC have been found to deteriorate their cognitive behavior in both males and females, but their BDNF concentrations are only decreased in males (Poulia et al., 2020). On the contrary, other studies show that a chronic and acute treatment of THC increases the concentration of BDNF and that it is correlated with enhanced cognitive capacity in male rats (Suliman et al., 2018). On the other hand, it has recently been documented that THC administered perinatally does not show alterations in basal dopamine levels but show alteration when the offspring are administered in acute doses, which could indicate a sensitization of THC administered maternally (Frau et al., 2019). As can be seen, the literature shows possible mechanisms without being conclusive about the physiological mechanisms of action of THC, therefore more studies are needed to evaluate the relationship between structure, function and behavior.

In summary, we conclude that the administration of THC in mothers produces a low magnitude effect on the behavior of the offspring. This is expressed in less activity relative to control subjects in several tasks. If we separate the effect on the types of behavior, we can say that THC affects cognitive behavior in a medium magnitude and low in locomotor and emotional behavior.

## Data availability statement

The raw data supporting the conclusions of this article will be made available by the authors, without undue reservation.

## Author contributions

SR, GM, LP, and ML contributed equally to the design, implementation, and writing of this work, while VQ-S, FA, and FV supervised the implementation of the study, contributed to its writing, and edited previous versions of the manuscript. All authors contributed to the article and approved the submitted version.
